# Association of Apolipoprotein E (APOE) Gene Polymorphism With Age-Related Macular Degeneration (AMD) in Indian Patients

**DOI:** 10.7759/cureus.85441

**Published:** 2025-06-05

**Authors:** Divya Gupta, Shobhit Chawla

**Affiliations:** 1 Biochemistry, People’s College of Medical Sciences and Research, Bhopal, IND; 2 Ophthalmology, Prakash Netra Kendra, Gomtinagar, Lucknow, IND

**Keywords:** amd, apoe, pcr, polymorphism, rflp, sequencing

## Abstract

Background

Age-related macular degeneration (AMD or ARMD; Online Mendelian Inheritance in Man (OMIM) #603075) is the degenerative disease of the retina that causes progressive impairment of central vision, leading to irreparable vision loss in older persons.

Objective

The objective was to investigate the association between apolipoprotein E (APOE) gene polymorphism and AMD in Indian patients.

Materials and methods

Genotyping for APOEvariants was performed in 121 AMD patients and 100 healthy controls using a polymerase chain reaction and restriction fragment length polymorphism (PCR-RFLP) method, followed by sequencing. Genotype and allele were analyzed using the allele counting method. P-value < 0.05 was considered statistically significant.

Results

The E3/E3 genotype has a frequency of 76/100 (76%) in controls and 97 (80%) in AMD. The frequency of the heterozygous E2/E2 genotype was 19 (19%) in controls and eight (6.6%) in AMD, and the variant E4/E4 genotype was found in one control (1%) and in one AMD case (1%). Some protective effect of the E2/E2 heterozygous genotype is seen (OR = 0.30; *P-*value = 0.009). Wild allele E3 in controls was 155 (77.5%) in AMD 209 (86.3%), E2 allele 41 (20.5%), and 17 (7%) in controls and AMD. E4 alleles in controls were four (2%) and 16 (6.6%) in AMD. E2 allele showed a protective effect; we did not find any significant association in APOE variant genotypes, and the odds ratios were within a 95% confidence interval (95% CI). As independent risk factors for AMD, logistic regression (LR) analysis was applied using E2, E3, and E4 alleles and their genotypes.

Conclusion

This study raises the possibility that the APOE gene might not have a significant association with AMD in Indian patients. However, our sample statistics suggest that the APOE E4 allele may be a risk factor for AMD in the Indian population. The study requires verification in a large sample size, across different parts of the country, and among other ethnic groups.

## Introduction

Age-related macular degeneration (AMD or ARMD; Online Mendelian Inheritance in Man (OMIM) #603075) is a neurodegenerative disease of the retina that causes progressive impairment of central vision and is the leading cause of irreversible vision loss in older people. Primarily, AMD affects the macular region, which contains a high density of photoreceptors that provide detailed central vision of the retina.

The first stage of the disease is called age-related maculopathy (ARM). Prevalence of AMD increases with age; it affects 65 years of the age 9% and 70 years of the population aged 30% of the population [1]. In India, the prevalence of AMD varies from 1.84% to 2.7%, as reported by different epidemiological studies [2]. A small number (1%) of the population has been reported to be ≥65 years of age. It is estimated that global AMD prevalence is exceeding and approximately 200 million people will be affected due to lack of therapies by the year 2040 [3].

This is a major problem due to irreversible vision loss in AMD. Therefore, etiology of disease is not known and is often regarded as a complex multifactorial disorder that has a profound effect on the environmental (smoking, diet, and sunlight exposure can impact the progression of AMD) and genetic risk factors genes related to the complement system and lipid metabolism, have been linked to an increased risk of AMD.

Apolipoprotein E (APOE; OMIM # 107741) is involved in the pathogenesis of AMD, and is also responsible for other age-related or increasing age or aging disorders such as Alzheimer’s disease (AD) and atherosclerosis [4]. APOE is a highly functional protein that is highly expressed in the liver, brain, and retina [5]. The etiology of APOE has been reported in specific diseases. AMD is mainly caused by abnormal metabolic processes, and lipid metabolism is one of the key elements of AMD. In lipid metabolism and lipid-associated metabolism, various enzymes have been involved, and they have also been found to be correlated with the initiation and advancement of AMD disease pathology. Numerous studies have reported that drusen anatomy and substructures include 30 different types of proteins (APOE and APOB), as well as esterified and non-esterified cholesterol, with a 3.2% dry weight of long fatty acids [6]. In lipid transportation and metabolism, APOE and other proteins are involved and have also been highly studied in AMD pathology. Due to its highly polymorphic nature, the APOE protein plays various important roles in lipid homeostasis and lipid metabolism, as well as in the central nervous system (CNS) [7].

Immuno-radiological studies show that the APOE gene of the eye is limited to common key components of the eye, such as retinal pigment epithelium (RPE), Bruch’s membrane, Müller cells, drusen, and basal deposits [4,5]. In the neural retina system, the role of microRNA (miRNA) in the APOE gene is synthesized by Müller cells and RPE [5]. A polymorphism in the APOE gene is a risk factor for several neurodegenerative disorders, and the APOE protein appears in the lesions of neurodegenerative disorders. The APOE gene is mainly involved in AMD pathogenesis, as it affects systemic cholesterol levels and has been shown by APOE gene knockout mice model data [8].

A study on a knockout mouse model showed that morphologic changes have occurred in the Bruch’s membrane of the APOE gene [9]. Several studies reported that the pathophysiology of AMD, such as deposition of sub-retinal drusen, reticular drusen, condensing of Bruch’s membrane, atrophy, hypo and hyper-pigmentation of RPE, is associated with APOE gene’s isoforms. Due to disease seriousness of the disease, AMD was found to be associated with APOE E4 (epsilon 4), high-expressing senescent mice, which were subjected to high-fat cholesterol [4]. Cell-membrane remodeling is an essential mechanism for the maintenance of the retina and its normal functions [4], which may be regulated by the APOE gene. Due to the advancement of age, lipids accumulate, which may lead to the formation of a hydrophobic barrier in the retinal Bruch’s membrane, which is responsible for causing AMD.

In general, APOE is expressed near the RPE, Müller glia cells, and is a profuse component of drusen in both AMD and non-AMD patients’ retina [5]. Both apical and basal surfaces of RPE are secreted by APOE and also contribute to the movement of lipid between cellular compartments, cell organelles, and the movement of lipid through Bruch’s membrane to the choriocapillaris by intracellular and extracellular [10]. It has been collected in the basal cytoplasm of RPE cells near the drusen, and this accumulation is mainly considered local APOE expression, resulting in a possible minor participation from extravasated particles from choroidal circulation [5]. The APOE gene, which functions as a cholesterol and lipid transporter, is a crucial component in drusen formation.

Studies based on mouse models provide awareness about the APOE gene function and its isoforms in the retina. In continuation, increased/high serum cholesterol levels in APOE knockout mice show condensed Bruch’s membrane with an orientation of Bowman’s membrane, abnormalities in retinal function as measured by electroretinography, and the accumulation of membrane-bounded material ultrastructurally comparable to that of blind individuals [9].

The APOE gene, located on chromosome 19q13.2, and the APOC-I/C-II gene complex are closely linked. The APOE gene contains four exons and three introns spanning 3597 base pairs (bp), and it produces a 299-amino-acid-long polypeptide protein chain. APOE is synthesized in various tissues and organs such as the brain, liver, spleen, kidneys, gonads, adrenals, and macrophages [7]. APOE type produces APOE (epsilon), and isoforms of APOE (epsilon) are encoded by epsilon 2 (E2), epsilon 3 (E3), and epsilon 4 (E4), which are known to be differentiated by the same amino acid found in the two main protein components. The combination of these three alleles produces six genotypes (E3/E3; E2/E2; E4/E4; E2/E3; E3/ E4; and E2/E4). Molecular basis of these polymorphisms is a misinterpretation of codons 112 (rs429358) and 158 (rs7412), i.e. APOE2 containing variation (Cys 112, Cys 158), APOE3 containing amino acid variation (Cys 112, Arg 158), and APOE4 containing amino acid variation (Arg 112, Arg 158) [11]. APOE3 is a very common allele. The allele frequency of E2, E3, and E4 is approximately 8%, 78%, and 15%, respectively, in Caucasian populations [12]. In humans the APOE gene allele frequency of E3 is approximately 50-90% and E2 and E4 vary between 5-35% and 1-15%, respectively, and their relationship with lipid levels E2 allele is associated with lower serum low-density lipoprotein (LDL), although published literature shows that, E4 allele is associated with higher LDL cholesterol [13]. It is the perception that the APOE4 allele is involved in injury, infection, platelet function, programmed cell death, and damage to cells and tissue. These isoforms vary greatly in terms of protein structure and function [14]. Available literature shows that the E4 isoform of the APOE gene is associated with a variety of neurodegenerative diseases, mostly from AD [14]. Associated with aging diseases, such as heart disease, AD, which share certain traits, are common in etiopathogenesis and AMD, APOE association with AMD, and then investigated [4,15]. Soft drusen is a hallmark of the early stage of AMD development and is also a ubiquitous component of the APOE gene [5]. Genetic association studies between APOE and AMD have been investigated multiple times, although the results have been inconsistent.

## Materials and methods

Patients and clinical examination

The study protocol was approved by the Institutional Ethics Committee (IEC). All subjects were considered for the study after undergoing a detailed ophthalmological examination, including visual acuity measurement, slit-lamp biomicroscopy, and dilated fundus examination, performed by an ophthalmologist and a retinal surgeon. Both dry and wet types of AMD were considered for the study after the fluorescein angiography. Control samples were collected after ophthalmological examination. The selection criteria were age of more than 70 years and clinically confirmed cases with no evidence of AMD. One hundred twenty-one clinically diagnosed AMD cases and 100 healthy control samples were collected after obtaining informed consent.

Molecular analysis

A 5.0 mL of venous blood was collected from all study subjects in an ethylenediaminetetraacetic acid (EDTA) vacutainer, and blood samples were stored at -80ºC. Genomic DNA isolation was performed by using a commercially available genomic DNA extraction kit (QIAamp Blood DNA Mini Kit, Qiagen, Hilden, Germany), according to the manufacturer’s protocol. Molecular analysis of the APOE gene was performed using polymerase chain reaction and restriction fragment length polymorphism (PCR-RFLP) and sequencing methods. For the APOE gene, a 244 bp DNA fragment was amplified using forward primer 5’-AGACGCGGGCACGGCTGTCCAAGGA-3’ and reverse primer 5’-CCCTCGCGGGCCCCGGCCTGGTACAC-3’ (NC_000019.10), which corresponds to the flanking regions covering amino acid positions 112 and 158 of exon 4. The PCR reaction mixture (GeNeiTM, Genei Laboratories Pvt. Ltd., Bengaluru, India) and PCR conditions of APOE gene polymorphism were as follows: buffer 10X with MgCl₂ 2.5 µL, Taq polymerase (1 U/µL) 1.5 µL, dNTPs (10 mM) 1 µL, forward primer (10 pmol) 1 µL, reverse primer (10 pmol) 1 µL, DNA 1.5 µL (50 ng/µL), and HPLC water 16.5 µL per 25 µL reaction. PCR conditions were as follows: hot lid 105ºC for five minutes, denaturation 95ºC for five minutes, 38 cycles for denaturation step at 94ºC for 45 seconds, 68ºC annealing for 45 seconds, and extension at 72ºC for 30 seconds, followed by final extension at 72ºC for five minutes and hold at 15ºC. The PCR-amplified product was 244 bp, and a product check was performed on 2% agarose gels pre-stained with ethidium bromide (EtBr), with a migrating distance of approximately 3 cm. The product bands were visualized under a UV transilluminator. The amplified PCR product (15 µL) was digested with HhaI (10 U) (New England Biolabs, Ipswich, MA) restriction enzymes at 37°C for 24 hours. The digested product was run on a 15% polyacrylamide gel electrophoresis (PAGE) for 10-12 hours at 80V in 1X tris-borate-EDTA buffer (TBE). After electrophoresis, the PAGE gel was stained with EtBr for 15 minutes, and the separated bands were visualized under a UV transilluminator and documented in the Molecular Imager Gel Doc XR System (Bio-Rad, Hercules, CA). After digestion, details of the products' band sizes are given in Table 1.

**Table 1:** PCR product size, restriction enzyme used, and after-digestion band size Note: Bands of sizes less than 38 bp ran away and were not visualized on the gel.

Polymorphism	PCR product	Enzyme	After digestion
APOE	244 bp	HhaI	Wild (E3/E3) - 91 + 48 + 38 + 33 + 18 + 16 bp. Heterozygous (E2/E2) -91 + 81 + 38 + 18 + 16 bp. Mutant (E4/E4) - 72 + 48 + 38 + 33 + 19 + 18 + 16 bp. E2/E3 - 91 + 81 + 48 + 38 + 33 + 18 + 16 bp. E3/E4 - 91 + 72 + 48 + 38 + 33 + 18 + 16 bp. E2/E4 - 91 + 81 + 72 + 48 + 38 + 33 + 19 + 18 + 16 bp

APOE genotypes give a restriction fragment pattern with continuous fragments of 38, 18, and 16 bp. The 18 bp and 16 bp fragments were of small size and distinctly detectable in all analyses. The APOE E2/E2 heterozygous genotype shows an extra 48 bp fragment, whereas the genotype APOE E3/E3 lacks the 83 bp fragment, which is formed, owing to the cleavage site at position 112 in the APOE E4 allele; 15% PAGE gel images were taken by the Molecular Imager Gel Doc XR System (Bio-Rad, Hercules, CA). Randomly, 10% of the samples were validated using the sequencing method with an ABI sequence analyzer (Applied Biosystems Inc., Foster City, CA), and the results were correlated with those of RFLP.

Statistical analysis

Allele and genotype frequencies were estimated using the allele counting method. Hardy-Weinberg estimates (HWE) for genotype frequencies were calculated using the Haploview software (Dr. Mark Daly's lab, MIT/Harvard Broad Institute, Cambridge, MA) that uses the expectation-maximization (EM) algorithm. HWE was tested by using the χ2 test with 1 degree of freedom, and statistical significance was considered if the p-value < 0.05. Calculating the odds ratio (OR) and its 95% confidence interval (CI) was performed. Logistic regression (LR) is used to adjust covariate effects by age and gender. All these calculations were performed by the commercial statistical analysis software SPSS (IBM SPSS Statistics for Windows, IBM Corp., Version 21, Armonk, NY).

For the APOE gene, LR analysis was performed with different APOE genotypes, as well as for E2, E3, and E4 alleles, which are considered independent components, with the risk of AMD being the dependent variable.

## Results

In this study, we recruited 121 AMD patients, comprising 74 males and 47 females, and 100 controls, including 71 males and 29 females without AMD, following detailed ophthalmological evaluation. The mean age was 70.40 years (60-96 years) and 74.42 years (70-95 years) for AMD patients and controls, respectively. Genotypes were successfully determined by PCR-RFLP, and 10% of the samples were further validated using the sequencing method. Genotype frequency of APOE gene showed in Table 2. Appendix A and Appendix B show the PAGE gel figure and sequencing chromatogram figures of APOE gene respectively.

Control samples were followed by the HWE (χ2 test with 1 degree of freedom). The APOE E3/E3 genotype was the most frequent genotype with a frequency of 76 (76%) in controls and 97 (80%) in AMD patients. The frequency of the APOE E2/E2 heterozygous genotype was 19 (19%) in controls and eight (6.6%) in AMD patients. For the variant APOE E4/E4 genotype, the frequencies were one (1%) in controls and one (1%) in AMD patients. Hence, on the basis of genotypes, no inference can be drawn due to the very few numbers of heterozygotes and homozygotes for APOE E2 and APOE E4 alleles. Some protective effect of APOE E2/E2 heterozygosity has been seen (OR = 0.30; *P*-value 0.009). The frequency of the APOE wild allele E3 in controls was 155 (77.5%), whereas in AMD cases, it was 209 (86.3%). For the E2 allele, the frequencies were 41 (20.5%) in controls and 17 (7%) in AMD patients. The frequency of the APOE variant allele E4 in control samples was 04 (2%) and in patients, 16 (6.6%). When we compared allele frequencies, the APOE E2 allele showed a protective impact. In contrast to published data from India, our study showed an odds ratio (OR) of the E4 allele about three-fold, but the *P*-value was greater than 0.05 with the χ^2^ test and 1 degree of freedom, suggesting a lack of statistical significance. After analyzing the APOE gene, genotypes, and allele frequencies, we did not find any meaningful correlation in APOE variant genotypes (E4/E4) when comparing them with AMD patients and controls, as shown in Table 2.

**Table 2:** Distribution of APOE gene polymorphisms among 121 AMD patients and 100 controls Odds ratios (ORs) were calculated and reported within the 95% confidence interval (CI) limits. Logistic regression (LR) analysis was performed using APOE alleles (E2, E3, and E4) and their genotypes as independent variables in relation to AMD. AMD, age-related macular degeneration; APOE, apolipoprotein E

Genotypes	AMD patients (n = 121)	Controls (n = 100)	p-value	OR (95% CI)
APOE genotype				
E2/E2	8 (6.6%)	19 (19%)	0.009	0.303 (0.123-0.744)
E2/E3	1 (8.2%)	2 (2%)	0.881	1.167 (0.153-8.917 )
E2/E4	0 (0%)	1 (1%)	NC	NC
E3/E3	97 (80%)	76 (76%)	Reference genotype	
E3/E4	14 (11.5%)	1 (1%)	0.018	12.965 (1.566-107.359)
E4/E4	1 (0.82%)	1 (1%)	0.772	0.661 (0.040-10.915)
APOE allele frequency				
E2	17 (7%)	41 (20.5%)	0.000	0.308 (0.168-0.5626)
E3	209 (86.3%)	155 (77.5%)	Reference allele	
E4	16 (6.6%)	4 (2%)	0.056	2.967 (0.973-9.048)

## Discussion

The aim of our study was to investigate the association of polymorphisms in the APOE gene with AMD in the Indian population. Although studies from various populations have been conducted, the results have been inconsistent and vary from disease to disease and population to population. The APOE E3 allele is the most common worldwide, and the frequencies of E2 and E4 vary significantly from population to population, with APOE E2 being absent in Native Americans [16]. Available literature shows that APOE E4 is the major genetic risk factor for AD and sets the stage for neuropathological disorders precipitated by genetic, metabolic, and environmental stressors. APOE also influences susceptibility to parasitic, bacterial, and viral infections. In HIV-positive patients, APOE E4 homozygosity is associated with a faster progression to AIDS and death and increases susceptibility to opportunistic infections. However, for AMD, the APOE E4 allele appears to provide protection. AD and other diseases for which APOE E4 is a risk factor are characterized by enhanced neurodegeneration and impaired brain plasticity repair [17]. Animal and cellular model studies revealed that APOE E4 is associated with impaired cellular plasticity [17,18]. Hence, the adverse effects of APOE E4 in AD are due to reduced neuronal synaptic plasticity, which subsequently leads to AMD, characterized by amplified angiogenesis and vascular plasticity. The role of APOE E4 could be protective because of the reduction in retinal pathological neovascularization [19].

Another study by Liu et al., on the Hakka population, has shown that the frequency of E3/E4 increased in type 2 diabetes mellitus (T2DM) patients with cardiovascular disease (CVD) (p < 0.01) [20]. The E4 allele was higher in CVD patients without T2DM (p < 0.01) and T2DM patients with CVD (p < 0.01) as compared with the controls [20]. The result of this study is consistent with published studies [21].

Studies on the Finnish population have proven that the prevalence of coronary artery disease (CAD) in diabetes patients varied according to types of APOE genotypes, which was 81% in E4 patients, 58% in E3/3 patients, and 53% in E2/2 or E2/3 patients [21]. However, the genotypes of APOE E4/4 and E4/3 are inclined to enhance the severity of macrovascular disease in T2DM patients [21].

Data on Indian AMD patients is limited; however, based on numerous studies, most have shown an association between the E2 allele and AMD, and the E4 allele as protective against AMD. In the study of Kaur et al., the study of 100 AMD patients, the E2 allele frequency was slightly higher in cases than in control subjects (0.04 vs. 0.03), whereas the E4 allele frequency was slightly higher in cases compared to controls (0.15 vs. 0.07), with no significant statistical differences [22]. In our study, there were a few E4/E4 and E2/E4 genotypes in both AMD cases and controls.

The OR of the E4 allele was three-fold higher compared to E3, but their p-value > 0.056, which shows a lack of statistical significance. The OR for E3E4 genotypes was 13, with a p-value of 0.018. This indicates that the APOE E4 allele increases susceptibility to AMD, though the frequency is low in the Indian population. It may be a significant contributing factor in some cases of AMD. The OR for the APOE E2 allele and E2/E2 genotype were 0.308 and 0.303 (*P*-value 0.009), respectively, suggesting a protective effect.

Although available literature from other populations reports an association between the E2 E4 genotype and AMD, the findings from the present study do not support it. This controversial finding is supported by available population data on the APOE polymorphism in the Indian population, where the E4 allele is more common compared to the E2 allele. A recently published study on the Northern Spanish Population showed that allele and genotype frequencies did not show significant differences as compared to the AMD cases and controls. The statistical analysis revealed by Fernandez-Vega et al. showed that APOE, E2E2 carrier genotypes were less frequent in AMD than in the controls [23]. They also found that the frequency of the E2 allele was higher in the control group than in the patients. In this study, the author revealed that the E2 allele showed a protective effect in AMD patients, and this finding is also supported by the current study data [23]. A study from India, conducted on patients with coronary heart disease (CHD) and controls, concluded that the APOE E4 allele is more prevalent in the case cohort, at 15.1% in patients and 6.7% in controls [24]. Similarly, Kharrazi et al. reported a comparable finding that APOE E4 allele frequency among CHD patients was higher (18.7%) than in controls (3.3%) [25]. A study from Hong Kong showed that the APOE E4 allele frequency was 12.6% and 7.6% in CHD cases and controls, respectively [26].

For normal function and maintenance of the retina, the APOE gene might play an important role in the remodeling of the cell membrane [4]. It might be possible that the ineffectiveness of APOE E4 to form dimers compared with APOE E2 or APOE E3 variants allows simple transport of lipid due to the small size of lipid particles through Bruch’s membrane [27]. Due to increased age, accumulation of neutral lipids may lead to the formation of hydrophobic barriers within Bruch’s membrane of the retina.

Anderson demonstrates that the genetics of the APOE E4 allele is linked to AMD, and the most important risk factor for developing AMD is an accumulation of drusen in the macula. They also showed that the cellular remainders derived from the RPE, deposited between the basal plate and Bruch’s membrane, contribute to a chronic inflammatory response, and following the drusen formation, this phenomenon has been supported by other studies also [5,28]. This loss of progressive sight in the central part of the macula contributes to local inflammation. Therefore, local inflammation on the macula contributes to drusen formation and also suggests that this process is similar to other age-related disorders or ageing diseases, such as AD and arteriosclerosis. Sanan et al. demonstrated that the APOE E4 isoform binds to beta amyloid protein more rapidly than the APOE E3 isoform [29]. A review article published by Hu et al. noted that the APOE E2 allele is associated with high risk and the APOE E4 allele is associated with low risk, in comparison to the APOE E3 allele [30]. APOE E4 presents a major genetic risk factor, with neuro-inflammation and amyloid-beta (β) deposition features that are common with AMD and also very common in age-related disorders such as AD. The complement system and amyloid-β are shown to have a possible relationship with the APOE gene, which is discussed here to provide information on their roles in the pathogenesis of AMD, biogenesis of drusen, immune cell activation and recruitment, and inflammation in the retina [30]. AMD sensitivity loci are still being searched for, and it is an important part of searching for the interaction between genetic and environmental factors. A comprehensible perception of AMD pathogenesis could be most readily accomplished through the acknowledgement of these complex interactions. However, further studies are required on larger sample sizes to reveal the possible role of the APOE gene in the pathogenesis of AMD in different parts of the country and other ethnic groups as well.

Limitations of the study

A large number of study samples is required to validate our results.

## Conclusions

This study raises the possibility that the APOE gene may not play a significant role in AMD among Indian patients and suggests that the APOE E4 allele can be a risk factor in the Indian population. Whereas the E2 allele, as well as the E2/E2 (heterozygous) genotype, shows a protective effect against AMD. Identification of individuals at risk could help to make strategies for prevention, early diagnosis, and management of the disease.

## Figures and Tables

**Figure 1:**
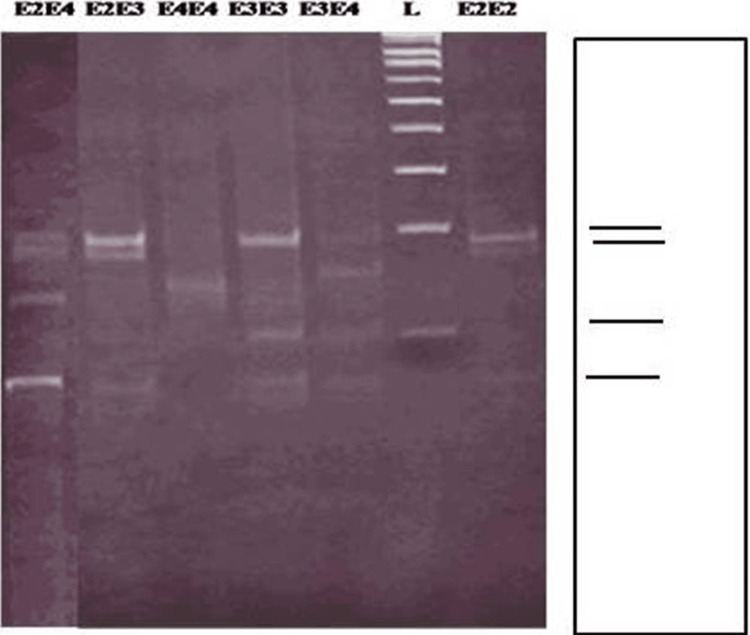
PCR-RFLP, PAGE gel figure of the APOE gene Typical gel picture of APOE genotyping: analysis of PCR amplified APOE gene digested with restriction enzyme HhaI, on a 15% polyacrylamide gel. Lane 1 E2E4, Lane 2 E2E3, Lane 3 E4E4, Lane 4 E3E3, Lane 5 E3E4, Lane 6 50 bp DNA ladder, and Lane 7 E2E2. PAGE, polyacrylamide gel electrophoresis; PCR-RFLP, polymerase chain reaction and restriction fragment length polymorphism

**Figure 2:**
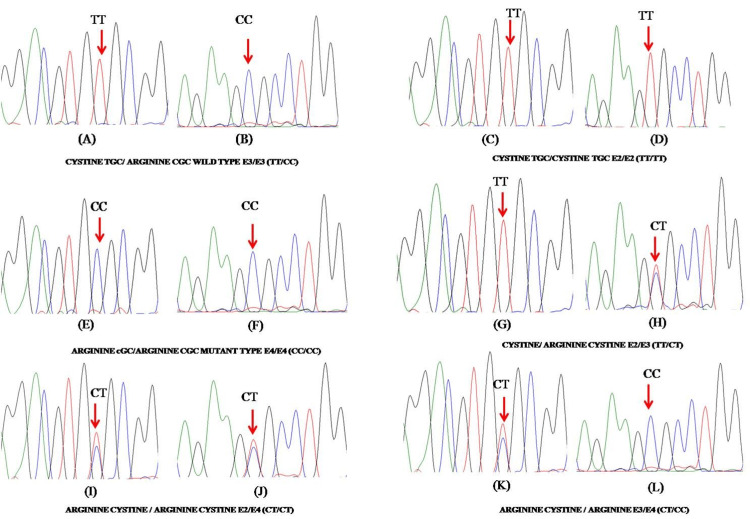
Sequencing chromatogram figures of the APOE gene Representative sequences for E2, E3, E4 alleles of APOE (A,B) E3/E3, (C,D) E2/E2, (E,F) E4/E4, (G,H) E2/E3, (I,J) E2/E4, (K,L) E3/E4. Note: E3 allele (wild type) as TGC sequence at codon 112 (cystine); CGC at codon 158; E2 allele has TGC at codon 158 instead of CGC. APOE, apolipoprotein E

## Appendices

Appendix A 

Appendix B 
